# A functional observational battery for evaluation of neurological outcomes in a rat model of acute bacterial meningitis

**DOI:** 10.1186/s40635-020-00331-1

**Published:** 2020-08-08

**Authors:** Jane Fisher, Chiara Pavan, Luisa S. Ohlmeier, Bo Nilson, Iben Lundgaard, Adam Linder, Peter Bentzer

**Affiliations:** 1 Faculty of Medicine, Department of Clinical Sciences Lund, Division of Infection Medicine, Lund University, Lund, Sweden; 2 Center for Translational Neuromedicine, Faculties of Health and Medical Sciences, University of Copenhagen, Copenhagen, Denmark; 3 Faculty of Medicine, Department of Laboratory Medicine, Division of Medical Microbiology Lund, Lund University, Lund, Sweden; 4 Clinical Microbiology, Labmedicin, Region Skåne, Lund, Sweden; 5 Department of Experimental Medical Science, University of Lund, Lund, Sweden; 6 Wallenberg Center for Molecular Medicine, University of Lund, Lund, Sweden; 7 Department of Anesthesia and Intensive Care, Helsingborg Hospital, Helsingborg, Sweden; 8 Division of Anesthesia and Intensive Care, Department of Clinical Sciences Lund, Lund University, Lund, Sweden

**Keywords:** Acute bacterial meningitis, Rat model, Neurological symptoms, Functional observational battery, *Streptococcus pneumoniae*, Pneumococcal meningitis

## Abstract

**Background:**

Acute bacterial meningitis is a disease with a high mortality and a high incidence of neurological sequelae in survivors. There is an acute need to develop new adjuvant therapies. To ensure that new therapies evaluated in animal models are translatable to humans, studies must evaluate clinically relevant and patient-important outcomes, including neurological symptoms and sequelae.

**Methods:**

We developed and tested a functional observational battery to quantify the severity of a variety of relevant neurological and clinical symptoms in a rat model of bacterial meningitis. The functional observational battery included symptoms relating to general clinical signs, gait and posture abnormalities, involuntary motor movements, focal neurological signs, and neuromotor abnormalities which were scored according to severity and summed to obtain a combined clinical and neurological score. To test the functional observational battery, adult Sprague-Dawley rats were infected by intracisternal injection of a clinical isolate of *Streptococcus pneumoniae*. Rats were evaluated for 6 days following the infection.

**Results:**

Pneumococcal meningitis was not lethal in this model; however, it induced severe neurological symptoms. Most common symptoms were hearing loss (75% of infected vs 0% of control rats; *p* = 0.0003), involuntary motor movements (75% of infected vs 0% of control rats; *p* = 0.0003), and gait and posture abnormality (67% of infected vs 0% of control rats; *p* = 0.0013). Infected rats had a higher combined score when determined by the functional observational battery than control rats at all time points (24 h 12.7 ± 4.0 vs 4.0 ± 2.0; 48 h 17.3 ± 7.1 vs 3.4 ± 1.8; 6 days 17.8 ± 7.4 vs 1.7 ± 2.4; *p* < 0.0001 for all).

**Conclusions:**

The functional observational battery described here detects clinically relevant neurological sequelae of bacterial meningitis and could be a useful tool when testing new therapeutics in rat models of meningitis.

## Introduction

Acute bacterial meningitis has a high mortality and high risk of neurological sequelae in survivors [1, 2]. *Streptococcus pneumoniae* (pneumococci) is the most common and deadly bacterial meningitis pathogen [3] and causes the most neurological sequelae in survivors [2]. The main empirical and directed treatment for bacterial meningitis is beta-lactam antibiotics [4], such as cefotaxime, ceftriaxone, and penicillin G, which cause lysis of bacteria [5]. These lysis products induce a strong immune response, leading to significant damage of the host tissues [6]. The only adjunctive therapy currently recommended for treatment of bacterial meningitis is corticosteroids, which can dampen this immune response [4, 7]. However, even in countries with widespread corticosteroid use, the rate of unfavorable outcome remains high at 38% [2] suggesting that new adjuvant therapies need to be explored.

Animal models are an important step in testing the efficacy of new therapeutics. Due to the broad range of possible symptoms and sequelae in bacterial meningitis [8], we suggest that a battery test that covers a broad range of symptoms should be considered for measurement of corresponding neurological outcomes in animal models. However, few studies using rat models attempt to quantify the wide range of possible neurological symptoms. The functional observational battery is typically used in neurotoxicology studies for identifying the neurological effects of new therapeutic compounds [9–11]. The International Conference on Harmonisation (ICH) S7A guideline requires the use of a battery test for testing the neurological effects of all new compounds before they can be administered in humans [12]. The adult rat is the recommended animal for evaluation of neurological symptoms in this guideline. The functional observational battery is therefore a well-accepted method for identifying neurological symptoms in rat models. To the best of our knowledge, the functional observational battery has not been used in animal models of bacterial meningitis.

Here, we developed a functional observational battery to include relevant symptoms of meningitis and assess its use in an adult rat model of pneumococcal meningitis.

## Methods

The local Ethical Committee for Animal Research has approved the experimental protocol (applications #143-16 and #13798/2018). We used 10-week-old male rats (Taconic). Animals were treated in accordance with the National Institutes of Health for the Care and Use for Laboratory animals, and Swedish legislation. We used 23 rats in total, eleven rats in the control group and twelve rats in the infected group.

### Preparation

We anaesthetized rats by intraperitoneal administration of pentobarbital (60 mg/kg; APL). Pentobarbital was chosen as the anaesthetic as it is metabolized slowly, ensuring that rats did not awaken during the preparation and infection procedure. The jugular vein was cannulated and the catheter tunneled to emerge from the back of the neck. The catheter was filled with 30 μL of heparin lock solution (5 Units/mL) to prevent coagulation and tied tightly.

### Bacterial culture

To ensure the clinical relevance of the model, we used a clinical isolate of pneumococci (serotype 6b) with intermediate sensitivity to penicillin previously isolated from a patient with bacteremia. We used a strain with intermediate sensitivity to antibiotics to increase the length of the infection, because we found that, when using a sensitive strain, 100% of bacteria were killed after only one dose of the appropriate antibiotic (data not shown). Bacteria were cultured as detailed in Supplementary file 1. We cultured the bacteria in 30 g/L Todd-Hewitt broth (Beckton-Dickinson), with 0.5% yeast extract (Oxoid), and 2% choline chloride (Sigma) as described in Supplementary method 1 to an optical density 620 nm (OD_620_) of 0.8. This solution was drop plated at several dilutions to check for bacterial viability in each batch. We found that the solution contained, on average, 3 × 10^8^ colony-forming units (CFU)/mL. The bacterial solution was kept on ice and used to infect rats within 4 h.

### Infection

We immobilized the rats on a stereotactic device and positioned them so that the nose was pointing downward to stretch the back of the neck. We exposed the cisterna magna and inserted a small needle (30G, SOPIRA), attached to flexible tubing, which we fixed temporarily to the skull with a drop of histoacryl (Braun) to prevent leakage and needle displacement. Then, we infused 20 μL of bacterial solution at a rate of 2 μL per minute. This corresponds to, on average, 6 × 10^5^ bacteria per injection. Control rats received an equal volume of 0.9% sodium chloride solution (Fresenius). After the infusion, the needle was left in the cisterna magna for 5 min to limit the backflow of the solution. We then removed the needle and sealed the hole with a drop of histoacryl and closed the incision.

We have observed that infected rats frequently develop secondary bacteremia and lung infections [13]. When housed in the same cage as infected rats, we observed that control rats often have developed bacteria in the lungs and display signs of lung infections after several days (data not shown). Therefore, infected and control rats had to be kept in separate cages.

### Drug dosing and administration

We applied a standard meningitis treatment of antibiotics (penicillin G; 43 mg/kg; Meda) and corticosteroids (betamethasone; 0.12 mg/kg; AlfaSigma) to both infected and uninfected rats. We administered this standard treatment to all rats in order to mimic the clinical standard treatment recommended for human bacterial meningitis [4, 7]. Several rat models described in the literature administer interventions as an adjuvant to antibiotics [14–18]. We suggest that meningitis models should include corticosteroids in addition to antibiotics because of their widespread use in human meningitis, and therefore, we chose to treat the rats in this study with both corticosteroids and antibiotics. The drugs were given every 12 h starting at 24 h after the infection (Table 1). Intraperitoneal injection of drugs was chosen because of the ease of administration and rapid transfer to the blood. If the model is to be used to test an intravenous adjuvant treatment that is incompatible with intraperitoneal injection, then the jugular catheter can be used to administer this treatment. In this case, the jugular catheter was used to administer two doses of 0.9% sodium chloride at 24 h and 36 h after the infection.

**Table 1:** Schedule of interventions and assessments

Time point							
	Day 0	Day 1		Day 2			Day 6
	0 h	24 h	36 h	48 h	60 h	*	144 h
	Infect	Treat and assess					Close-out
Preparatory surgery and infection	**X**						
**Interventions**							
Penicillin G + betamethasone		**X**	**X**	**X**	**X**	*	
**Assessments**							
Weight	**X**	**X**	**X**	**X**	**X**	*	**X**
Temperature	**X**	**X**		**X**			**X**
Functional observational battery		**X**		**X**			**X**
**Sample collection**							
CSF		**X**		**X**			**X**
Brain							**X**

*Every 12 h thereafter until close-out

### Evaluation

The intervention and assessment schedule is summarized in Table 1. All assessments and sample collections described in Table 1 were carried out in all rats. CSF sample collection was always done after all other assessments. As an ethical endpoint, three termination criteria were evaluated at least four times per day after the infection. Termination criteria included severe cramps, severe breathing difficulty, and lack of movement on provocation. Rats exhibiting decreased grooming were evaluated more frequently for these criteria as this indicates a high level of illness.

Weight was measured at baseline and every 12 h after the start of treatment. Temperature, neurological and clinical score, and activity score were measured as described above. Temperature was assessed before infection using a rectal thermometer. All measurements were always assessed prior to administration of drugs or collection of samples. Because infected and control rats had to be kept in separate cages, the assessor was not blinded to the infection status in this study.

### Functional observational battery

We adapted the functional observational battery from Moser et al. [19] and altered to include symptoms and terminology relevant to meningitis. Our functional observational battery (Table 2) measured clinical symptoms to yield a clinical score and neurological symptoms to yield a neurological score. The two scores were summed to yield a combined score. Neurological symptoms were subdivided into four categories: gait and posture abnormalities, involuntary motor movements, focal neurological signs, and neuromotor abnormalities. Negative geotaxis has previously been used in rat meningitis models [20, 21] and therefore has been added to the battery as a test of neuromotor abnormality. The details of each parameter, and their corresponding symptoms in human meningitis, are described below.

**Table 2:** Functional observational battery of neurological and clinical signs

	Score assigned			
	0	1 (slight)	2 (moderate)	3 (severe)
**Clinical signs**				
Breathing difficulty	Normal	Slightly shallow, heavy, or fast	Moderate difficulty	Severe difficulty (terminate the rat)
Movement upon provocation	Normal	Slightly lower than normal	Lower than normal	Extremely low or no movement; comatose (terminate the rat)
Grooming	Normal	Some dirt in fur	Dirt and staining of fur	Extremely dirty, matted fur
Vocalizing	None	Occasional vocalizing when handled	Some vocalizing (when handled or not)	Much vocalizing, even when not handled
Porphyrin accumulation	Absent	Small amount around eyes or nose	Clearly present around eyes or nose	Severe accumulation around eyes or nose
Piloerection	Absent	Slight	Moderate	Severe
Clouded eyes	Absent	Slight (one or both eyes)	Moderate (one or both eyes)	Clouding completely obscures pupil in one or both eyes
Exophthalmia	Absent	Slight (one or both eyes)	Moderate (one or both eyes)	Severe (one or both eyes)
**Neurological signs**				
Gait and posture				
Ataxia	Absent	Slight	Moderate	Severe
Hindlimbs	Normal movement and positioning when walking	Slightly abnormal movements or positioning	Abnormal movement or positioning; weakness apparent; may be walking on toes	Severe abnormality, severe weakness, paresis
Forelimbs	Normal movement and positioning	Slightly abnormal movements or positioning	Abnormal movement or positioning; weakness apparent; may be walking on toes	Severe abnormality, severe weakness, paresis
Body positioning	Normal, pelvis kept off the floor	Slightly flattened pelvis when sitting or walking	Moderately flattened pelvis	Severely flattened, difficulty lifting body from floor
Spine curvature	Normal	Slightly hunched	Moderately hunched	Severely hunched
Involuntary motor movements				
Tremors	Absent	Slight tremors, may be present only during certain tasks such as reaching	Moderate intensity and persistence of tremors	Severe, constant tremors both when moving and at rest, large muscle groups affected (e.g. head)
Muscle jerks and spasms	Absent	Occasional small jerks or spasms	Jerks or spasms with moderate frequency or intensity	Severe jerks or spasms with high frequency or intensity
Tonic movements	Absent	Slight, occasional	Moderate, frequent	Severe, very frequent
Stereotypy	Absent	1 point for each stereotyped behaviour observed		
Bizarre behaviours	Absent	1 point for each bizarre behaviour observed		
Focal neurological signs				
Observational tests				
Increased lacrimation	Normal	Slight	Moderate	Severe
Increased salivation	Normal	Slight	Moderate	Severe
Eyelid drooping or closure	Absent	Slight in one or both eyes	Moderate in one or both eyes	Severe, one or both eyes completely closed
Vibrissae whisking	Normal	Slightly reduced on either side	Low movement on either side	No movement on either side
Manipulative tests				
Pupil reaction	Normal	Normal sized pupils with slightly slowed or low response	Normal sized pupils but no or very low response	Pupil has abnormal size (fully dilated or constricted) and no response
Blink reflex	Normal	Attempted response but incomplete closure of either eyelid	Attempted response but no closure of eyelid	No attempt at closure
Pinna reflex	Normal	x	No response	x
Hearing loss	Normal	x	Delayed or very weak response (e.g. ears give only a small twitch)	No auditory startle response
Neuromotor tests				
Negative geotaxis response	Normal positioning in < 6 s	Normal positioning in 6–30 s	Normal positioning in > 30 s	No attempt to reposition or slides down
Righting reaction	Normal positioning in < 3 s	Normal positioning in 4–30 s or abnormal placement of limbs during repositioning	Normal positioning in > 30 s	No attempt to reposition

To limit the effect of environmental variation on the behaviour of the rats, the same assessor always carried out the observation in the same location with the same light and sound level, at the same time of day (morning). Most characteristics were assessed while the rat was in an open field consisting of a box with dimensions 49.5 × 27 cm covered with a plastic paper at the bottom. A fresh paper cover was used for each rat to limit olfactory cues that could influence their behaviour.

#### Clinical signs

The clinical signs assessed are described in Table 2. Many of these signs are specific to rats or rodents. Signs such as ungroomed fur, low movement upon provocation, vocalizing, porphyrin accumulation, and piloerection are generally accepted as signs of malaise or distress in rats [19, 22] and are not specific to meningitis. Breathing difficulty, clouded eyes, and exophthalmia (bulging eyes) are also signs of general malaise [19, 22], but might indicate secondary infections in the lungs or the eyes. Breathing difficulty and movement upon provocation were also evaluated as termination criteria, and if either of them were judged to be severe (a score of 3), the rat would be terminated. The presence of dirty, ungroomed fur was an indication to evaluate the rats more frequently.

#### Gait and postural characteristics

To assess gait and posture, rats were observed in the open field as described above. If needed, the rat was encouraged to walk by gentle prodding by the assessor, and the rats were scored according to Table 2. Gait and posture abnormalities in rodents are sensitive indicators of overall neurological dysfunction [19]. Gait and posture are controlled by several neurological pathways, and abnormalities can be caused by damage to the cerebral cortex, cerebellum, basal ganglia, and the vestibular system [23]. Abnormalities in gait and posture can also be due to paresis of the limbs resulting from focal neurological deficits. Gait was defined as the movements of the limbs as the rat moved around the field. The normal rat moves opposing front and back legs at the same time to remain upright [19]. Posture was defined as body placement and spine curvature. The normal rat should walk with its body held off the floor and its spine straight [19].

#### Involuntary motor movements

To assess involuntary motor movements, rats were observed in the open field described above and scored according to Table 2. Involuntary motor movements include tremors, muscle jerks and spasms, tonic movements, stereotypy, and bizarre behaviours. Involuntary motor movements can be caused by damage in several brain areas, most often the basal ganglia [24]. Stereotypies are normal behaviours that are performed repetitively and purposelessly, for example, pacing, head weaving, and persistent grooming. Although stereotypies are not typically reported in humans following meningitis, they are a common sign of distress in laboratory animals [25, 26] and can be indicative of inappropriate responses in the basal ganglia [26]. Other bizarre behaviours such as self-mutilation, odd tail positioning, and teeth grinding, which do not fit into the above categories, were also recorded as they could indicate other neurological or clinical problems.

#### Focal neurological signs

To assess focal neurological signs, rats were observed in the open field described above. Focal neurological signs were scored according to Table 2. Focal neurological signs are common during and after bacterial meningitis and are defined as a deficit localized to a specific site in the central nervous system, leading to loss of function localized to a specific area of the body [27]. In meningitis, lesions leading to focal neurological deficits can be caused by cerebral infarctions and damage due to increased intracranial pressure [8].

We chose to assess focal neurological signs that were easy to observe in rats while also being relevant to human bacterial meningitis. Hearing loss is the most common neurological sequela in bacterial meningitis [8]. Palsies of the cranial nerves, especially nerves III, IV, VI, and VII, are seen in 10–20% of meningitis patients [28]. We assessed palsies of cranial nerve III (oculomotor nerve) [29] by assessing eyelid drooping and pupillary reaction. We assessed palsies of cranial nerve VII (facial nerve) [30] by assessing lacrimation, salivation, blink reflex, and pinna (ear) reflex. Vibrissae (whisker) whisking movements are not relevant to humans, but are often used in rats as a measure of cranial nerve VII function [31, 32], and therefore, this was also included in the assessment.

*Lacrimation* (tear production) and *salivation* (saliva production) abnormality were noted if obvious wetness was observed around the eyes and around the mouth, respectively. The basal level of lacrimation and salivation in rats is low [19]; therefore, it was only possible to assess increases in these characteristics.

The *pupil reaction* was tested by first observing the size of the pupils, and then gently covering the rat’s head with a dark cloth. Upon removal of the cloth, a normal rat should exhibit dilated pupils that constrict rapidly (within seconds) to their original size [19].

The *palpebral* (*blink*) *reflex* was tested by slowly bringing the edge of a fine wire from the nose toward the nasal point of each eye. In a normal rat, the eyelids should close quickly upon approaching the eye [19].

The *pinna* (*ear*) *reflex* was tested by gently touching a fine wire to the skin or hair inside the ear. In a normal rat, the ear should shake or flatten upon light contact [19]. Because a severity of response is difficult to assess for this measure, the pinna reflex was scored only as “present” or “absent”. An absent reflex was scored as 2 points.

*Hearing loss* was assessed using a click test to induce an auditory startle response. The auditory startle response is a motor reflex that occurs in response to a sudden sound stimulus above 80 dB [33]. Severe hearing loss in humans is defined as a hearing threshold of 80 dB [34], corresponding to a complete loss of serviceable hearing [35]. Complete loss of the startle response would therefore indicate severe hearing loss, although it might also be caused by focal neurological damage of the response pathway itself [33]. A reduced or delayed startle response (e.g. only small ear movement in response) could indicate mild to moderate hearing loss or neurological damage to the response pathway.

To elicit an auditory startle response, a clicker was placed outside of the open field, and outside of the rat’s field of vision, approximately 15 cm away from the rat. The assessor used the clicker to make a single loud click. The healthy rat immediately moves its ears, flinches, or startles in response to the sound [19, 33]. A complete absence of response was given a score of 3, corresponding to a “severe” grade in the other tests. If the rat gave a delayed or very small response, indicating acknowledgement of the sound but no startle, this was given a score of 2, corresponding to a “moderate” grade in the other tests. A score of 1 was not used for this test.

#### Neuromuscular and neuromotor tests

Neuromuscular and neuromotor tests were scored according to Table 2. These tests were used to evaluate overall muscle coordination, strength, and vestibular and sensory function [19]. These tests included a negative geotaxis response and a righting reaction.

*Negative geotaxis response* is a postural reflex that tests motor function and vestibular function [36]. Rats were placed head-downward on a plane inclined at an angle of 30°. The plane was covered in a rough wood surface to allow rats to grip the surface. Normal adult rats exhibit a negative geotaxis response in which they turn their body 180° and face their head up the slope [36]. The response normally occurs within seconds. The amount of time it took for the rats to complete this movement was noted and scored according to Table 2.

A *righting reaction* was used to test vestibular function, coordination, and strength [19]. The rat was placed on its back on the bottom surface of the open field box. The rat was quickly released, and the time, limb position, and body positioning during its return to a standing position were noted. The healthy rat should flip immediately to a standing position, first turning its head, then forelimbs, and finally its hindlimbs [19].

### Collection of samples

#### CSF collection

Cerebrospinal fluid (CSF) was collected 24 h, 48 h, and 6 days after infection. Isofluorane was chosen as the anaesthetic at this step because it is metabolized quickly and the rats could awaken soon after the procedure was complete. Rats were anaesthetized with isofluorane gas (5% induction and 2.5% maintenance, AbbVie), and the cisterna magna was exposed as described above. The wound was always cleaned with chlorhexidine which was allowed to evaporate. Then, the cisterna magna was punctured with a 33-G needle, and at least 20 μL of CSF was aspirated. Samples were kept on ice until further processing.

#### Brain collection

When rats were sacrificed, they were decapitated and the brain removed and cut in half. The hemispheres were immersion fixed in phosphate-buffered saline (PBS; Sigma Aldrich) with 4% paraformaldehyde (PFA; Sigma Aldrich) overnight.

### Laboratory analysis

#### CSF bacterial load

CSF samples were diluted by factors of 10, 100, 1000, and 10,000 using sterile PBS. Four replicates of 20 μL of each dilution were plated on blood agar plates using the drop plate method, whereby the liquid was dispensed as a drop onto one side of a blood agar plate. The plate was then tilted to allow the drop to roll down to the other end of the plate, leaving a streak of each solution. Plates were incubated at 37 °C overnight (at least 14 h). Identity of pneumococcal colonies was confirmed by observing an alpha-hemolytic zone surrounding the colonies. The number of colony-forming units (CFU) of pneumococci in each 20 μL streak was counted, and the amount of CFU per millilitre of CSF was calculated. For each sample, the lowest dilution factor in which individual colonies were not overlapping and were easily separated by eye, but which had at least 10 colonies per streak, was used for the calculation.

#### Immunocytochemistry of brain samples

Drop fixed brains were kept in 4% PFA overnight at room temperature and then placed in PBS solution and stored at 4 °C until processing. Brains were embedded in 1.5% agarose (A5093, Sigma Aldrich) diluted in PBS and sectioned using a vibratome (100 μm sections; Leica VT1200S). Free-floating sections were washed 3 times in PBS, blocked for 2 h at room temperature in blocking solution containing 0.3% Triton X-100 (Sigma Aldrich), and 5% normal goat serum (Gibco™; Thermo Fisher Scientific) in PBS. The tissues were incubated at 4 °C overnight with primary antibodies diluted in blocking solution. Primary antibodies used in this study were mouse anti-myeloperoxidase (MPO; 1:100; NBP1-51148, Novusbio), rabbit anti-ionized calcium-binding adapter molecule 1 (Iba1; 1:500; 019-19741, Wako), and rabbit anti-active Caspase-3 (1:500; ab49822, Abcam). We chose Caspase-3 as an apoptosis marker because it is the major effector in most apoptosis pathways, and therefore, the presence of active Caspase-3 is a strong indicator of apoptosis, although a lack of active Caspase-3 does not necessarily rule out the activation of alternative apoptosis pathways [37]. The following day, tissues were incubated with appropriate secondary antibodies corresponding to the primary antibody species, conjugated to Alexa Fluor (AF) fluorophores at 1:500 dilution in blocking solution for 2 h at room temperature. Secondary antibodies used were goat anti-mouse AF-488, and goat anti-rabbit AF-568 (Thermo Fisher Scientific). After three PBS washes, tissues were counterstained with DAPI (4′,6-diamidino-2-phenylindole; 1:1000; 1 μg/mL; 62248, Thermo Fisher Scientific) for 10 min at room temperature, then mounted with ProLong® Gold Antifade Mountant (P36934, Thermo Fisher Scientific).

#### Microscope imaging

Images of the immunolabelled slides were acquired using a confocal microscope (Nikon Eclipse Ti) with Plan Fluor 20x 0.75 numerical aperture (NA), oil objective. Images were acquired at constant exposure levels throughout the study.

### Statistical analysis

The data were analysed per-protocol using the statistical software GraphPad Prism version 8. Quintile-quintile (QQ) plots were visually examined to determine whether data was normally distributed. The Shapiro-Wilk test for normality was used to confirm this assessment. We found that most data were normally distributed except for bacterial load. Therefore, parametric tests were used in all cases except those involving bacterial load.

Outcomes that were measured at multiple time points in each rat, namely activity score, neurological and clinical scores, weight loss, and temperature were compared between infected and control rats at each measured time point using a repeated measures 2-way analysis of variance (ANOVA) with Geisser-Greenhouse correction for sphericity and Sidak’s post hoc test for multiple comparisons. Neurological and clinical scores were also summed to yield a combined score that was treated the same way.

Incidence of neurological outcomes was calculated by determining the number of rats exhibiting any symptoms with at least moderate severity in each category (gait and posture, involuntary motor movements, hearing loss, other focal neurological signs, and neuromotor impairment) at 6 days after the infection. Hearing loss was analysed separately from other focal neurological signs because it is the most common neurological sequela in meningitis patients. Incidence of neurological symptoms was compared in infected and control rats using Fisher’s exact test. Because bacterial load was not normally distributed, the correlation between bacterial load on day 1 with neurological score on day 6 in infected rats was determined using a Spearman correlation.

## Results

### Clinical characteristics

**Fig. 1.**
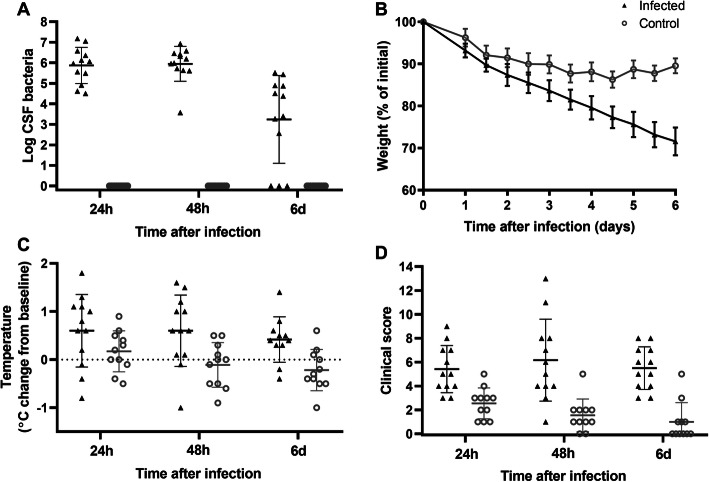
Clinical characteristics of infected rats and saline control rats. Rats were infected with pneumococci (infected; black triangles; *n* = 12) or with equal volume of saline (control; grey circles; *n* = 11). The following outcomes are reported: **a** log-transformed CSF bacterial load, **b** weight as a percentage of the initial weight on day 0, **c** change in temperature from baseline, and **d** clinical score as measured by the functional observational battery. Lines and error bars indicate the mean and standard deviation

Rats were evaluated for clinical signs of illness. No rats died in either the infected or the control group. Control rats had no detectable pneumococcal colonies in the CSF (Fig. 1a); however, they did lose weight for the first 48 h and had a mean clinical score of 2.5 ± 1.3 at 24 h after intracisternal infusion (Fig. 1b, c). This effect was likely due to a reaction to the preparatory surgery and injection procedure. The mean clinical score in control rats decreased to 1.0 ± 1.6 by day 6, suggesting that repeated cisterna magna puncture and CSF collection likely did not increase clinical symptoms over time.

Infected rats had a mean bacterial load in CSF of 5.0 ± 3.1 × 10^6^ CFU/mL at 24 h after the infection (Fig. 1a), confirming the presence of bacterial meningitis. Infected rats lost more weight than control rats (Fig. 1b), losing a mean of 28.5 ± 3.3% of their body weight by day 6 compared to a loss of 10.4 ± 1.8% in the control group (*p* < 0.0001). Temperature change from baseline (before infection) was calculated by subtracting the baseline temperature for each rat. Infected rats had significantly elevated temperature compared to controls (Fig. 1c) at 48 h (0.60 ± 0.74 vs 0.17 ± 0.42 °C above baseline; *p* = 0.013) and 6 days (0.42 ± 0.47 vs − 0.22 ± 0.43 °C above baseline; *p* = 0.030). Infected rats also had a significantly increased clinical score (Fig. 1d) compared to control rats at all time points (24 h 5.4 ± 2.0 vs 2.5 ± 1.3, *p* = 0.0016; 48 h 6.2 ± 3.4 vs 1.5 ± 1.4, *p* = 0.0020; 6 days 5.5 ± 1.8 vs 1.0 ± 1.6, *p* < 0.0001).

**Fig. 2:**
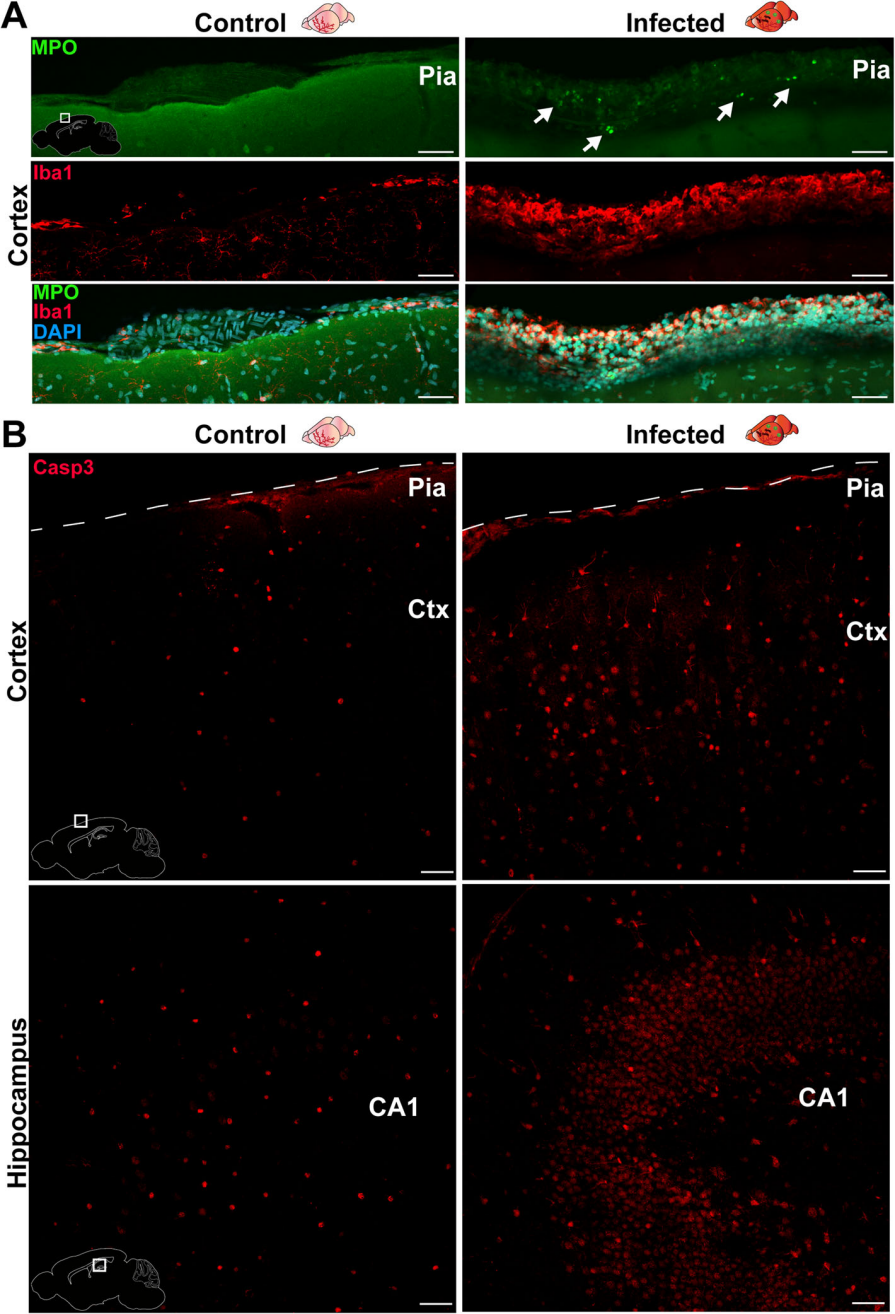
Brains of infected and control rats. Rats were infected with pneumococci (infected) or with equal volume of saline (control). **a** Brain sections were stained for neutrophils (MPO, green), macrophages/microglia (Iba1, red), and DNA (DAPI, blue). An area of the cortex featuring the meningeal space was imaged. White arrows indicate MPO-positive cells (neutrophils). **b** Brain sections were stained for active Caspase-3, a marker of apoptosis (Casp3, red). An area of the cortex (Ctx, top; dashed line indicates the pia membrane) and the hippocampus (CA1, bottom) was imaged. Scale bars (white horizontal lines) are 50 μm. White schematic insets depict the imaged brain region in each case

To further confirm that rats had cellular changes in the brain consistent with bacterial meningitis, rat brain slices from one rat with a median neurological score and from one saline control rat were stained for MPO, a neutrophil marker, and Iba-1, a macrophage/microglia marker (Fig. 2a), and a section of the meninges was imaged. Neutrophil and macrophage/microglia cell infiltration was found in the meninges of the infected rat, but not the saline control rat. Brains of these rats were also stained for active caspase-3 (a marker of apoptosis), and the cortex and hippocampus were imaged (Fig. 2b). A greater amount of caspase staining was found in both brain regions in the infected rat than in the control rat, indicating the presence of apoptotic cells in the infected rat. Caspase staining was stronger in the cortex than in the hippocampus, as expected in adult rats with meningitis [38].

### Neurological symptoms

The majority of infected rats exhibited neurological symptoms with a severity of 2 or more, when scored using the functional observational battery (Table 3). Hearing loss, involuntary motor movements, and gait and posture abnormalities were the most frequently observed symptoms. No control rats had any neurological symptoms with a severity of 2 or more. Infected rats had a significantly higher incidence of hearing loss (*p* = 0.0003), other focal neurological signs (*p* = 0.0046), gait and posture abnormality (*p* = 0.0391), and involuntary motor movements (*p* = 0.0001) than control rats. Involuntary motor movements were primarily tremors and muscle jerks. No rats exhibited movements that would be consistent with severe clonic-tonic seizures; however, some involuntary motor movements observed (facial muscle movements, head bobbing) are included on the Racine scale for seizures in rats [39], so the presence of seizures during observation, and seizures occurring outside of the evaluation time, could not be ruled out.

**Table 3:** Incidence of any neurological symptoms with score ≥ 2 at day 6

	Infected (***n*** = 12)	Control (***n*** = 11)	***p*** value
Hearing loss; *n* (%)	9 (75%)	0 (0%)	0.0003
Focal neurological signs excluding hearing loss; *n* (%)	6 (50%)	0 (0%)	0.0137
Gait and posture abnormality; *n* (%)	8 (67%)	0 (0%)	0.0013
Involuntary motor movements; *n* (%)	9 (75%)	0 (0%)	0.0003
Neuromotor impairment; *n* (%)	2 (17%)	0 (0%)	0.4783

**Fig. 3:**
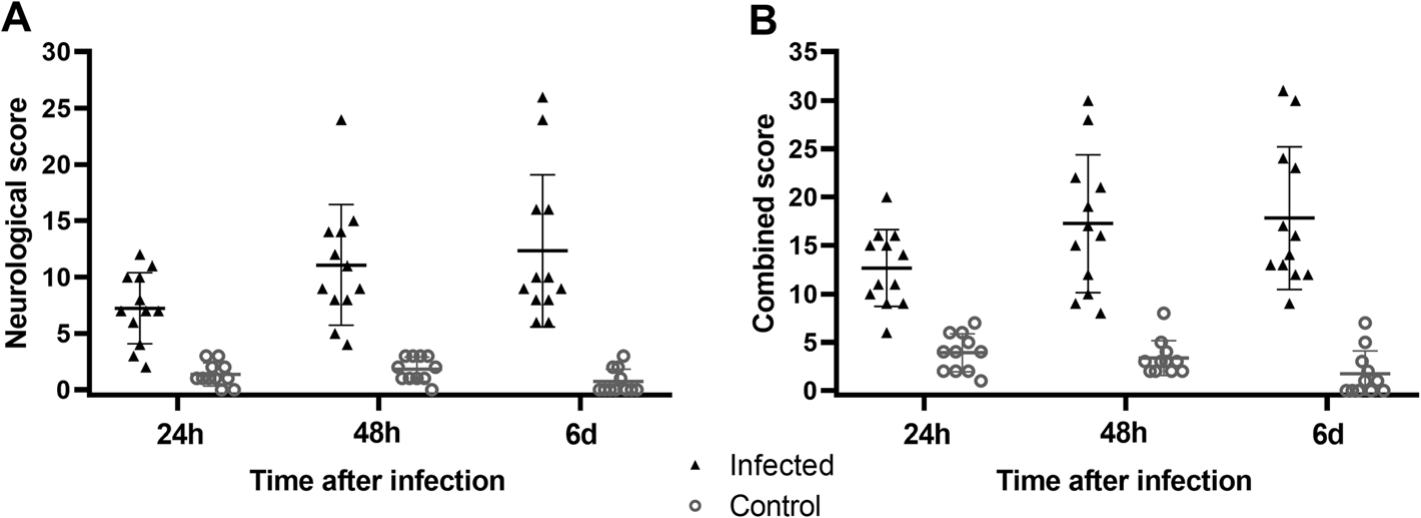
Neurological score of infected rats and saline control rats. Rats were infected with pneumococci (infected; black triangles; *n* = 12) or with equal volume of saline (control; grey circles; *n* = 11). The following outcomes are reported: **a** neurological score as measured by the functional observational battery and **b** combined clinical and neurological score as measured by the functional observational battery. Lines and error bars indicate the mean and standard deviation

Neurological score (Fig. 3a) was significantly higher in infected rats compared to controls at all time points (24 h 7.3 ± 3.2 vs 1.4 ± 1.0, *p* < 0.0001; 48 h 11.1 ± 5.4 vs 1.8 ± 1.1, *p* = 0.0002; 6 days 12.3 ± 6.7 vs 0.73 ± 1.1, *p* = 0.0003). A combined score (Fig. 3b), obtained by summing the clinical and neurological scores, was also significantly higher in infected rats compared to controls at all time points (24 h 12.7 ± 4.0 vs 4.0 ± 2.0; 48 h 17.3 ± 7.1 vs 3.4 ± 1.8; 6 days 17.8 ± 7.4 vs 1.7 ± 2.4; *p* < 0.0001 for all). A Spearman correlation revealed that, in infected animals, bacterial load 24 h after the infection was significantly correlated with neurological score on day 6 (Spearman *R* = 0.776, *p* = 0.0042).

## Discussion

In this study, we have explored the use of an adapted functional observational battery to quantify a wide range of neurological symptoms of bacterial meningitis in a rat model. We found that the functional observational battery was able to detect clinically relevant symptoms of bacterial meningitis.

When testing new therapeutics in animal models, it is important to include patient-important outcomes, such as neurological symptoms and sequelae. Few reported rat models of bacterial meningitis examine functional measures of neurological symptoms. Some measure only a few specific symptoms such as hearing loss [40–42], cognitive deficits [43, 44], or depressive-like behaviours [45]. While these measurements are valuable for evaluating specific symptoms, they do not take into account the wide range of possible symptoms of meningitis. This could easily lead to reporting bias where only the tests with positive results are reported. Other reported models use scoring systems featuring a 4- or 5-point scale to evaluate the righting reaction and activity level [46–48]. The most comprehensive of the reported scoring systems evaluates seven categories of symptoms, but misses many of those included in the functional observational battery [49]. We suggest that the functional observational battery is valuable because it takes into account a wider range of potential symptoms than other methods.

In our meningitis-adapted version of the functional observational battery, we used a 3-point scale to evaluate 8 clinical symptoms and 20 individual neurological symptoms in five categories. Infected rats had a clear increase in both scores compared to controls. However, by day 6, the range of neurological scores was quite broad. Sprague-Dawley rats are out-bred, and the different genetic backgrounds could result in different abilities to clear the infection. Indeed, we found a clear correlation of bacterial load on day 1 with neurological symptoms on day 6. The wide range of severity and types of neurological symptoms in our model is reminiscent of meningitis in humans. Patients with bacterial meningitis typically present with heterogeneous symptoms, and evaluation of individual symptoms has a poor diagnostic ability [50]. Our functional observational battery easily captures the heterogeneity of neurological symptoms of bacterial meningitis.

The functional observational battery identified neurological symptoms that are relevant to human bacterial meningitis. Hearing loss is the most common neurological sequela in humans and develops within the first few days of the course of illness, affecting 22–69% of adults with pneumococcal meningitis [8]. In our rat model, we detected hearing loss in 75% of rats by day 6. Focal neurological deficits occur in 11–36% of adults with pneumococcal meningitis, typically developing during the course of illness [8]. Focal neurological signs can cover a wide range of symptoms [27]. Although we only included 8 signs that were easily evaluated in rats, mostly having to do with cranial nerve palsies, we found that 50% of rats developed these focal neurological signs by day 6. The functional observational battery therefore appears to be well suited to quantify both the incidence and the severity of relevant neurological symptoms of bacterial meningitis.

In this study, a single observer made all measurements, and therefore, we did not conduct any measures of inter-observer variability. If a study is carried out by more than one observer, then it is important to provide some measure of inter-observer variability [51]. Measurements made by different observers can be affected by experience and training [51], and rat behaviour can be affected by individual characteristics such as the sex of the observer [52]. Therefore, it is generally recommended that all tests in a study involving a functional observational battery are conducted by the same observer throughout the study to reduce the effects of this individual variation [19]. If the measurements cannot be conducted by a single observer, then various statistics can be used to check that their observations are in agreement, such as Kappa statistics [53]. The same rats should be scored by all observers in a random order to ensure independence of the observations. The handling and testing procedure itself may induce stress [19] or the rats may become habituated to some stimuli [33], so measurements should be separated in time or limited in frequency to limit variation due to these effects.

A strength of this study is that we adapted a well-established method of neurological evaluation, the functional observational battery, and carefully considered the relevance of each symptom for bacterial meningitis. The functional observational battery can easily be adapted to include other symptoms such as memory deficits or depressive-like behaviours. A limitation of the study is the fact that we did not randomize individuals into infected and control groups because the risk of cross-infections required keeping *S. pneumoniae-*infected rats in separate cages. This would make any attempted blinding of the assessor easy to break, and so the assessor was not blinded in this study. This effect has potential repercussions for other animal models of meningitis and implies that the unit of randomization should be by cages and not by individual animals as is often the case. Another limitation is that we only tested the functional observational battery in male rats. Although there are well-documented gender differences in the response to infection in humans and in animal models [54], most animal models of meningitis use only male rats and so we included only males in this proof of concept study.

## Conclusions

We have found that a functional observational battery was a relatively rapid and sensitive method for quantification of a wide range of relevant neurologic symptoms of bacterial meningitis in a rat model. We suggest that a comprehensive scoring system, such as a functional observational battery, should be added to animal models of bacterial meningitis when testing new therapeutics in order to evaluate their overall neurological effects.

## Supplementary information

**Additional file 1: Supplementary method 1.** Growth of pneumococci.

## Data Availability

The datasets used and/or analysed during the current study are available from the corresponding author on reasonable request.

## Abbreviations

AF: Alexa Fluor
ANOVA: Analysis of variance
CFU: Colony-forming units
CSF: Cerebrospinal fluid
DAPI: 4′,6-Diamidino-2-phenylindole
Iba1: Ionized calcium-binding adapter molecule 1
ICH: International Conference on Harmonisation
MPO: Myeloperoxidase
NA: Numerical aperture
PBS: Phosphate-buffered saline
PFA: Paraformaldehyde
QQ: Quintile-quintile
