# Supplementary material for: A functional observational battery for evaluation of neurological outcomes in a rat model of acute bacterial meningitis
Source: Intensive Care Med Exp. 2020 Aug 8;8:40. doi: 10.1186/s40635-020-00331-1 (PMC7415049; doi:10.1186/s40635-020-00331-1)
Supplement: Supplementary file 1 — Additional file 1: Supplementary method 1. Growth of pneumococci. [file 40635_2020_331_MOESM1_ESM.docx]

**Growth of pneumococci**

*Plating*

The bacteria were stored in a stock solution in 40 % glycerol at -80 °C. We have observed that freeze-thaw cycles decrease bacterial viability, and therefore we did not allow the stocks to thaw when used. We used a 1μL inoculation loop to scoop a small amount of the frozen stock solution and streaked it onto a pre-warmed blood agar plate. We incubated the plate at 37°C for 18-20 hours.

*Liquid culture*

To ensure that we could obtain a sufficient amount of bacteria, we scraped all colonies from the plate into a tube of pre-warmed Todd-Hewitt broth (30g/L, brand), with 0.5% yeast extract (brand), and 2% choline chloride (Sigma). Because pneumococci begin an autolysis process when the bacterial number is high [1], it is important to ensure that the starting concentration is not too high. Therefore we recorded the initial optical density (OD) of the solution at 620nm and adjusted the solution to below 0.2 OD_620_. We have observed that poor growth occurs when the culture is agitated by mixing and shaking. Therefore, we incubated the culture at 37°C without shaking, with the lid loosened to allow for aeration. The culture was mixed by gentle inversion every 30 minutes.

*Growth*

We have found that pneumococci follow a specific growth curve (supplementary figure 1), with a lag phase that lasts approximately 2 hours followed by a logarithmic growth phase that continues until the bacteria reach approximately 0.3-0.4 OD_620_ (4-5 hours). After this point we have observed that growth slows considerably, likely due to autolysis. The long lag phase likely makes pneumococcal cultures very sensitive to contamination with other faster-growing bacterial species. A growth curve that lacks this lag phase is therefore indicative of a contaminated culture. Therefore we checked the OD_620_ of the tube at least every hour to ensure that the growth was normal. Any cultures exhibiting abnormal growth not following this curve were assumed to be contaminated and discarded.

Once we observed that the culture was in the log-phase, but before beginning autolysis (after 4-5 hours of growth), we centrifuged the bacterial solution at 3000 x g for 10 minutes. We washed the resulting pellet once with normal saline and then re-suspended in normal saline to a final OD620 of 0.8. We drop-plated serial dilutions of this final solution to check the bacterial viability in each batch. The bacterial solution was kept on ice and used within 4 hours.


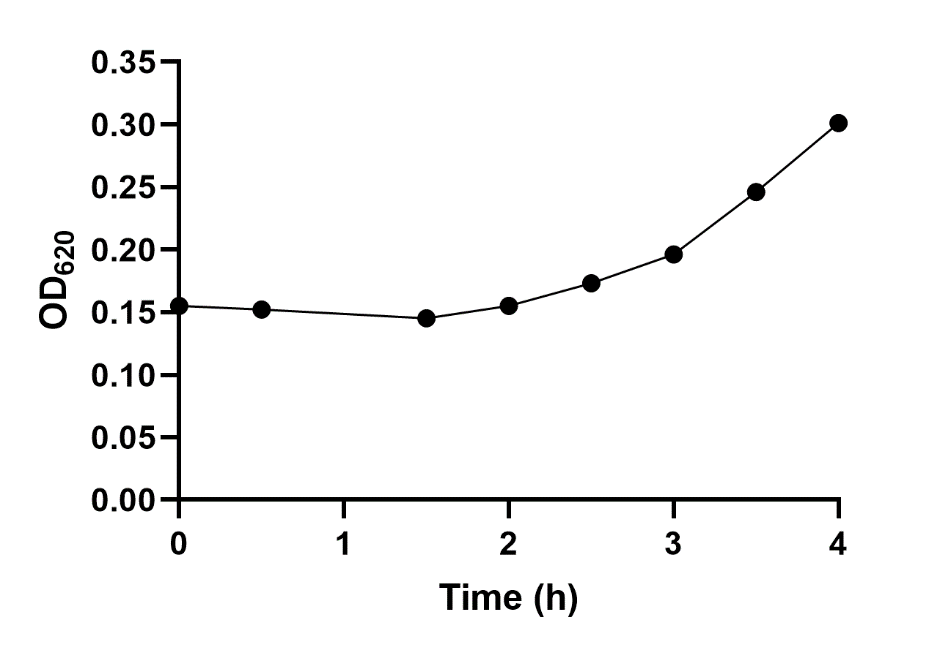


**Supplementary figure 1.** Representative growth curve of a pneumococcal culture grown at the conditions described above.

**References**

1. Restrepo A V, Salazar BE, Agudelo M, et al (2005) Optimization of culture conditions to obtain maximal growth of penicillin-resistant Streptococcus pneumoniae. BMC Microbiol 5:34
